# Surface scanning for 3D dose calculation in intraoperative electron radiation therapy

**DOI:** 10.1186/s13014-018-1181-0

**Published:** 2018-12-07

**Authors:** Verónica García-Vázquez, Begoña Sesé-Lucio, Felipe A. Calvo, Juan J. Vaquero, Manuel Desco, Javier Pascau

**Affiliations:** 1 Hospital General Universitario Gregorio Marañón, Unidad de Medicina y Cirugía Experimental (Instituto de Investigación Sanitaria Gregorio Marañón), Madrid, Spain; 2 Departamento de Oncología, Hospital General Universitario Gregorio Marañón, Madrid, Spain; 3 Facultad de Medicina, Universidad Complutense de Madrid, Madrid, Spain; 4 Clínica Universidad de Navarra, Madrid, Spain; 5 Departamento de Bioingeniería e Ingeniería Aeroespacial, Universidad Carlos III de Madrid, Madrid, Spain; 6 Centro de Investigación Biomédica en Red de Salud Mental (CIBERSAM), Madrid, Spain; 7 Centro Nacional de Investigaciones Cardiovasculares Carlos III (CNIC), Madrid, Spain

**Keywords:** IOERT, Intraoperative radiotherapy, Surface scanning, Conoscopic holography, Structured-light 3D scanner, Dose distribution

## Abstract

**Background:**

Dose calculations in intraoperative electron radiation therapy (IOERT) rely on the conventional assumption of water-equivalent tissues at the applicator end, which defines a flat irradiation surface. However, the shape of the irradiation surface modifies the dose distribution. Our study explores, for the first time, the use of surface scanning methods for three-dimensional dose calculation of IOERT.

**Methods:**

Two different three-dimensional scanning technologies were evaluated in a simulated IOERT scenario: a tracked conoscopic holography sensor (ConoProbe) and a structured-light three-dimensional scanner (Artec). Dose distributions obtained from computed tomography studies of the surgical field (gold standard) were compared with those calculated under the conventional assumption or from pseudo-computed tomography studies based on surfaces.

**Results:**

In the simulated IOERT scenario, the conventional assumption led to an average gamma pass rate of 39.9% for dose values greater than 10% (two configurations, with and without blood in the surgical field). Results improved when considering surfaces in the dose calculation (88.5% for ConoProbe and 92.9% for Artec).

**Conclusions:**

More accurate three-dimensional dose distributions were obtained when considering surfaces in the dose calculation of the simulated surgical field. The structured-light three-dimensional scanner provided the best results in terms of dose distributions. The findings obtained in this specific experimental setup warrant further research on surface scanning in the IOERT context owing to the clinical interest of improving the documentation of the actual IOERT scenario.

## Background

Intraoperative electron radiation therapy (IOERT) refers to the delivery of a single-fraction, high-energy electron beam during surgery with the goal of promoting local cancer control [1–3]. The target volume (namely a post-resection tumour bed or the macroscopic residue after partial resection) is irradiated by using a specific applicator that pushes aside healthy tissues and collimates the electron beam generated by a linear accelerator (LINAC) [1, 4, 5].

IOERT dosimetry relies on the conventional assumption of water-equivalent tissues in both stopping and scattering power at the applicator end, which defines a flat irradiation surface. Previous literature reported small differences between prescribed doses under previous assumption and measured doses using in-vivo dosimetry in the case of breast IOERT scenarios [6, 7]. However, Costa et al. [8], by placing radiochromic films on irradiation surfaces, showed that clinical two-dimensional (2D) dose distributions in pelvic IOERT scenarios frequently differ from the expected ones. In a previous study, Costa et al. [9] simulated these IOERT scenarios with solid water slabs and a radiotherapy bolus, highlighting that the shape of the irradiation surface (namely step-like or curved surfaces) modified the dose distribution. Documentation of the actual scenario is relevant to the quality assurance of IOERT and the proper assessment of clinical results. However, the applicator position, the angle of beam incidence in relation to the patient’s anatomy and the shape of the irradiation surface are not available in IOERT records. Intraoperative computed tomography (CT) studies with the applicator placed on the tumour bed would allow the calculation of three-dimensional (3D) dose distributions of the actual scenario before irradiation since these images include tissue heterogeneities, surface irregularities of the irradiated volume and the air gap from the applicator end to the tumour bed. A portable CT scanner inside the operating room or a LINAC with on-board kV cone beam CT may be used for that purpose [10]. However, a low number of IOERT interventions per week may not support the installation costs of in-room imaging devices [11]. In addition, intraoperative CT studies with metal artefacts (for example, owing to shielding discs and surgical retractors) cannot be directly used for dose calculation since CT values are substantially altered.

An intermediate approach between the IOERT conventional assumption and the use of intraoperative CT images would involve 3D scanning the surface of the tumour bed and the inclusion of these data in the dose calculation. In a preliminary study [12], the authors evaluated four different technologies to scan surfaces, selecting a custom-made structured-light 3D scanner as the most appropriate for IOERT in terms of resolution, accuracy, acquisition time and cost. However, this statement was based on scanning a half body mannequin, not simulating an actual IOERT scenario and calculating dose distributions. Brudfors et al. [13] presented an open-source software for scanning surfaces with a tracked conoscopic holography sensor that could also be used in IOERT. This system has been previously evaluated for clinical usage (for example, to scan human cadaver kidneys [14, 15] and resection cavities during neurosurgeries [16]).

In this study we aimed to explore, for the first time, the use of surface scanning methods for 3D dose calculation in IOERT. Two different 3D scanning technologies were evaluated in a simulated IOERT scenario, a tracked conoscopic holography sensor and a structured-light 3D scanner.

## Methods

In this section, we describe the 3D scanning systems assessed (section 3D scanning systems), the process of creating 3D images from surfaces (section Surface to pseudo-CT study for IOERT dose calculation) and the experiment designed to evaluate these non-contact devices for 3D dose calculation in IOERT (section Experiment). A simulated surgical field was scanned with both 3D scanning systems. 3D dose distributions obtained from CT studies of the surgical field (gold standard) were compared with those calculated under the conventional assumption of water-equivalent tissues at the applicator end and with those calculated from pseudo-CT studies based on surfaces.

### 3D scanning systems

#### Tracked conoscopic holography sensor

A conoscopic holography sensor measures distances to objects with an interferometric technique based on the double refraction of uniaxial crystals [17]. The collinearity between the laser beam emitted and the cone of light returned from the scanned object enables measurements of sharp edges and inside narrow cavities. Distances are converted into 3D coordinates of the scanned surface by tracking the sensor [14].

The configuration selected in this study was that presented in [13]: a ConoProbe Mark 10 sensor (Optimet, Optical Metrology Ltd) (working distance from 155 mm to 336 mm with the 250-mm objective lens as the origin, laser wavelength 655 nm, laser spot size 107 μm, laser power lower than 1 mW and weight 0.72 kg [18]) was tracked by a multi-camera optical tracking system OptiTrack (NaturalPoint, Inc.) with three optical cameras FLEX:V100R2 (data rate 50 Hz).

The data acquired with the tracked ConoProbe sensor (abbreviated hereafter to “ConoProbe”) is an unorganised point set that is converted to a triangle mesh by calculating its oriented surface normals and applying Poisson surface reconstruction [19] by means of open-source software MeshLab [20].

#### Structured-light 3D scanner

This 3D scanning technology is based on an incoherent light source that projects structured 2D patterns onto the scanned object, and a camera at a different viewpoint that records the patterns geometrically distorted by the object surface.

The specifications of the handheld 3D scanner selected in this study, Artec Eva (abbreviated hereafter to “Artec”), are: 3D resolution up to 0.5 mm, working distance from 0.4 m to 1 m, frame rate up to 16 fps and weight 0.85 kg [21]. This solution includes another camera to capture colour texture. The software Artec Studio 10 manages the capturing process, data post-processing, 3D model (triangle mesh) creation and texture mapping.

### Surface to pseudo-CT study for IOERT dose calculation

Dose distributions are calculated with a treatment planning system (TPS) specifically developed for IOERT (radiance, GMV) [22, 23] based on a Monte Carlo algorithm that takes account of the LINAC phase space [24] and tissue heterogeneities [25] (data obtained from CT studies after converting Hounsfield units (HU) values to physical density [10, 25]).

**Fig. 1:**
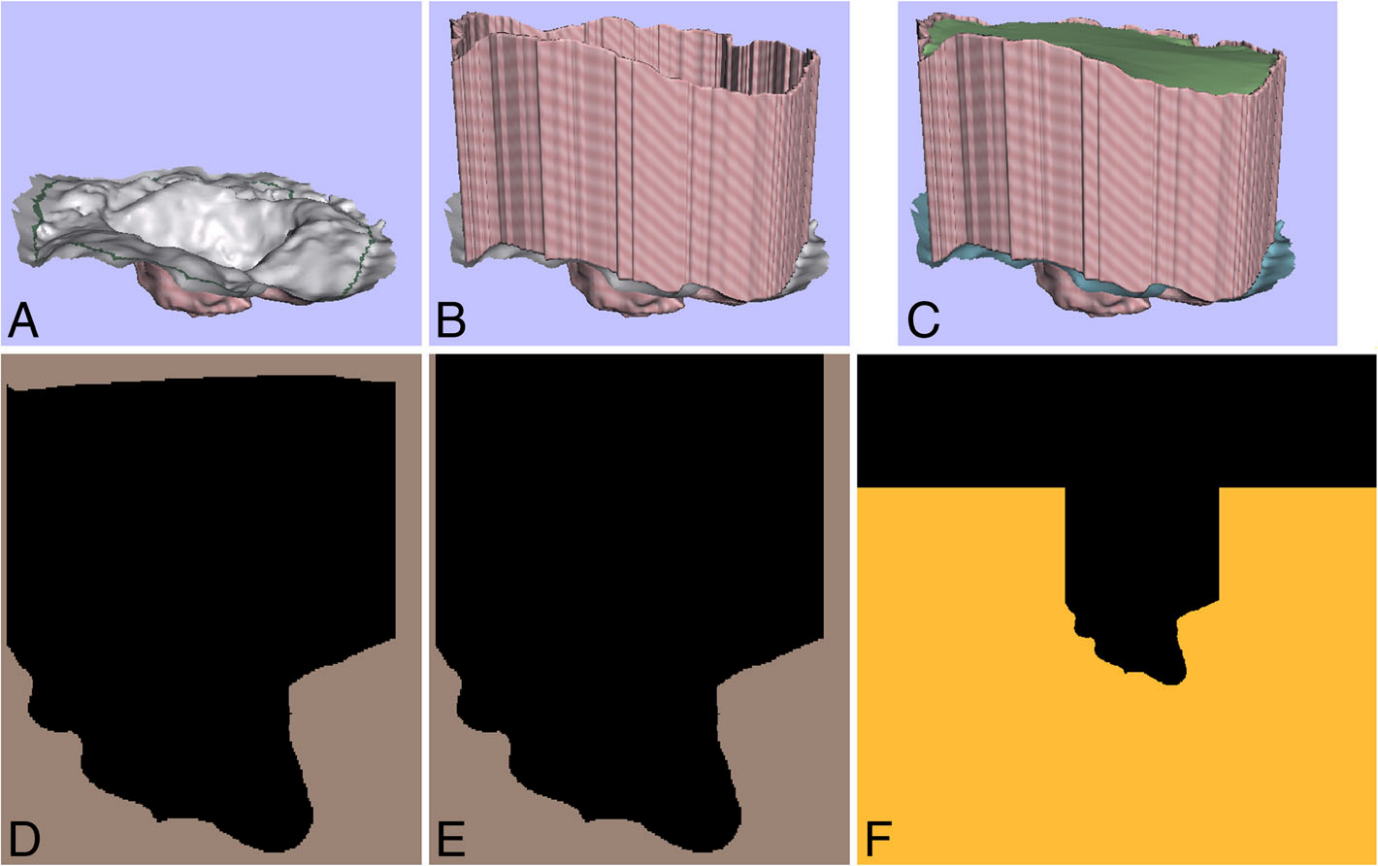
Conversion from 3D surface into pseudo-CT study. (**a**) 3D surface with a green contour delineated close to the mesh edge. (**b**) Extra walls generated after extruding the contour close to the mesh edge. (**c**) Green lid covering those extra walls to obtain a watertight surface. (**d**) Axial view of the 3D binary image generated after applying the ray intersection method to the watertight surface. The matrix size of this 3D image is limited to the boundaries of the watertight surface. (**e**) Axial view of the 3D binary image after correcting the region above the lid. (**f**) Axial view of the pseudo-CT study obtained by increasing the matrix size of the 3D binary image, and then by setting the *black* voxels to the CT value of air and the remaining voxels to the CT value of water (orange colour in the pseudo-CT study)

3D surfaces are converted into pseudo-CT studies for dose calculation. In this process (Fig. 1), we first generate a watertight surface by extruding a contour close to the mesh edge (out of the surgical field) and covering the extra walls generated with a lid (Autodesk software Meshmixer [26] and Artec Studio 10). Second, the watertight surface is converted to a 3D binary image by applying a ray intersection method [27] similar to that presented in [28]. Then, this 3D image is transformed into a pseudo-CT study (defined as a 3D image not acquired in a CT scanner but with values in the HU scale) by setting the voxels above the scanned surface to the CT value of air (− 1000 HU) and the remaining voxels to water value (0 HU).

### Experiment

#### Experiment set-up

**Fig. 2:**
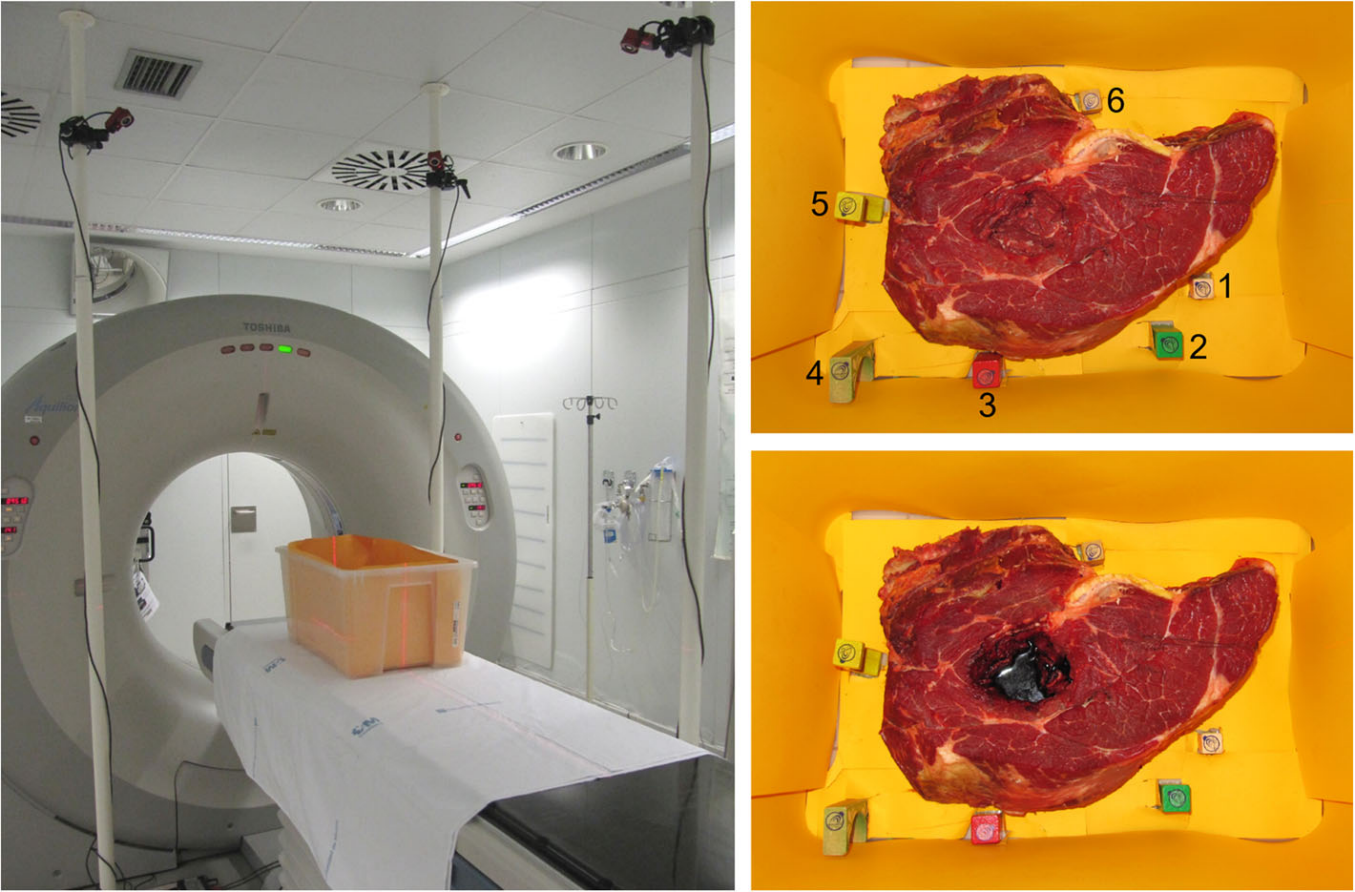
Experiment set-up. On the left, the CT simulator room with the multi-camera optical tracking system and the plastic box, which contained the simulated surgical field and the markers, on the CT table. The three optical cameras of the tracking system were attached to three posts. On the right, the simulated surgical field and the six markers placed at different heights. The configuration without blood is shown at the top right while the configuration with blood is depicted at the bottom right

The experiment was carried out in a CT simulator room in order to acquire reference CT studies. The cameras of the optical tracking system were statically attached to three STOLMEN posts (Inter IKEA Systems B.V.) around the table of an Aquilion™ Large Bore CT simulator (Toshiba) (Fig. 2).

A surgical field was simulated with a large piece of beef (weight 6 kg) placed at the bottom of a plastic box (volume 28 × 39 × 57 cm), on the CT table, as shown in Fig. 2. This large container simulated the volume of a sterile field with surgical retractors that may hinder 3D scanning. Six metallic nipple markers (diameter 1 mm, SL-10, The Suremark Company) were placed on cuboids of different heights around the phantom (Fig. 2). These markers were used as landmarks for mapping surfaces and CT studies into a common coordinate space.

#### Acquisition protocol

The details of the acquisition protocol are as follows:

1. A CT study of the surgical field and the markers (*CTpreSurf* or CT study acquired before surface scanning) was acquired with the following parameters: voltage 120 kVp, exposure 37 ± 10 mAs (mean ± standard deviation), matrix size 512 × 512 × 961 and voxel size 0.729 × 0.729 × 0.500 mm.
2. Laser beam (ConoProbe) sweeping of the surgical field and the markers (Fig. 2, top right) (minimum signal-to-noise ratio set to 40%).
3. Surface scanning of the surgical field and the markers (Fig. 2, top right) with Artec.
  Steps 2 and 3 were carried out from only one viewpoint to simulate the difficulty of moving around the patient in the operating room with our wired 3D scanners connected to both the power supply and the computer.
4. Step 1 was repeated with the same initial position of the patient table to obtain *CTpostSurf* (CT study acquired after surface scanning), which was used to check the stability of the whole setting (surgical field and markers) by comparing *CTpostSurf* and *CTpreSurf*. This verification was done because the surface irregularities of the simulated surgical field and the position of the markers could vary in steps 2 and 3 from the original setting shown in *CTpreSurf* due to the force of gravity, the movements of the CT table after *CTpreSurf* acquisition or a wrong attachment of the markers. The comparison was performed by superimposing *CTpostSurf* to *CTpreSurf* and calculating the root-mean-square error [RMSE] between the intensities from both studies without applying any alignment.
5. Steps 1 through 4 were repeated after adding 40 ml of pig blood with heparin (anticoagulant) to the surgical field (Fig. 2, bottom right) in order to simulate accumulation of biological fluid before irradiation.

#### Surface registration

Scanned surfaces were mapped to *CTpreSurf* to set a common coordinate space for comparisons in each configuration (with and without blood). The transformation matrix was calculated with a landmark rigid registration algorithm based on singular value decomposition [29] and the 3D coordinates of each marker (its centroid in the CT study and a point on its 3D surface) using 3D Slicer software [30] and SlicerIGT extension [31]. Colour texture (visible light picture data from the surface) was essential for obtaining the marker location in the Artec surfaces since the markers were not properly identified in the triangle meshes.

#### IOERT dose distributions

Registered surfaces were converted to pseudo-CT studies of 1-mm isotropic voxel size by applying the method described in section Surface to pseudo-CT study for IOERT dose calculation (obtaining *CTConoProbe* and *CTArtec* for ConoProbe and Artec surfaces respectively). *CTpreSurf* studies were also resampled to that voxel size.

An IOERT case was simulated in the TPS by placing a virtual applicator on *CTpreSurf*, *CTConoProbe* and *CTArtec* (parameters: applicator diameter 70 mm, bevel angle 30° and electron energy 9 MeV). The following 3D dose distributions were calculated for each configuration (with and without blood) with a Monte Carlo algorithm (error tolerance 1% [parameter that limits the maximum estimated uncertainty of the simulated dose for doses higher than 50% of the maximum dose] and resolution 1.0 mm) and the phase space of a conventional LINAC (Varian 21EX):

- *D_CTpreSurf* (considered as the gold standard) was calculated with *CTpreSurf*.
- *D_water* was calculated with *CTpreSurf* after selecting *Water* option in the TPS to obtain the dose distribution under the conventional assumption of water-equivalent tissues at the applicator end. *D_water* was obtained to assess the dose deviation when not including tissue heterogeneities, surface irregularities of the irradiated volume and the air gap from the applicator end to the tumour bed in the dose calculation.
- *D_CTtissue*&*air* was calculated after converting *CTpreSurf* to another pseudo-CT study (*CTtissue*&*air*) that had only two different CT values (namely water [0 HU] and air [− 1000 HU]). *D_CTtissue*&*air* was used to assess the dose deviation when applying the same simplification of *CTConoProbe* and *CTArtec* in the dose calculation (namely not taking account of tissue heterogeneities but including surface irregularities of the irradiated volume and the air gap from the applicator end to the tumour bed). *CTtissue*&*air* was obtained by first segmenting the air in *CTpreSurf* using intensity thresholding method (maximum limit − 500 HU), and then setting those voxels to the CT value of air and the remaining voxels (specifically, tissue) to the CT value of water.
- *D_CTConoProbe* and *D_CTArtec* were calculated with *CTConoProbe* and *CTArtec* respectively.

The output of the Monte Carlo algorithm was a 3D dose distribution in percentage where 100% corresponded to the maximum dose along the clinical axis measured in a water phantom for the selected applicator diameter and energy of the electron beam (in this study, 70 mm and 9 MeV respectively), the bevel angle 0°, and the source-to-surface distance set when conducting the measurements for modelling the LINAC.

*D_water*, *D_CTtissue*&*air*, *D_CTConoProbe* and *D_CTArtec* were compared with *D_CTpreSurf* using global normalisation [32], and a 3D gamma criteria of 3%/3 mm (thresholds accepted clinically) for dose values greater than 10% or 70% (to focus on high-dose regions) [10]. 3D gamma analyses did not take account of dose differences in voxels not belonging to tissue in *CTpreSurf*.

#### Evaluation of “Surface to pseudo-CT study” conversion process

A source of error when using surface scanning methods for 3D IOERT dose calculation is the conversion from a 3D surface into a pseudo-CT study. The surface created to assess this process was *reproSurf*, which was created by first segmenting the voxels with the CT value of water in *CTtissue*&*air* (configuration without blood), then generating a watertight surface (both steps done with 3D Slicer software), and finally selecting a non-manifold surface of the surgical field like that obtained with ConoProbe and Artec (step done with Meshmixer software). The configuration chosen was that without blood since its surface was more irregular than that of the configuration with blood. A pseudo-CT study (*CTreproSurf*) was generated by applying the method detailed in section Surface to pseudo-CT study for IOERT dose calculation to *reproSurf*.

The evaluation consisted in comparing the 3D dose distribution calculated with *CTreproSurf* (*D_CTreproSurf*) and the reference 3D dose distribution *D_CTtissue*&*air*, which was calculated with the CT study used to generate *reproSurf*. *CTreproSurf* included both the error of generating the surface from *CTtissue*&*air* and the error when converting that surface into a pseudo-CT study. *D_CTreproSurf* was obtained with the same IOERT parameters detailed in section IOERT dose distributions and both dose distributions were compared using the same method described in section IOERT dose distributions. This comparison enabled us to assess the influence of the conversion from a 3D surface into a pseudo-CT study on the dose calculation.

Table 1 summarises the image studies and 3D dose distributions previously explained in sections “Acquisition protocol”, “IOERT dose distributions” and “Evaluation of 'Surface to pseudo-CT study' conversion process”. Throughout the article, the prefix “CT” refers to the CT/pseudo-CT study and the prefix “D_CT” refers to the 3D dose distribution calculated from the CT/pseudo-CT study.

**Table 1:** Summary of CT/pseudo-CT studies and 3D dose distributions obtained in the experiment

	CT acquisition before surface scanning^a^	Surface scan with Conoprobe^a^	Surface scan with Artec^a^	CT acquisition after surface scanning^a^	Surface from *CTpreSurf* ^b^
Generated 3D images	*CTpreSurf* *CTtissue&air*	*CTConoProbe*	*CTArtec*	*CTpostSurf*	*CTreproSurf*
3D dose distributions	*D_CTpreSurf* *D_water* *D_CTtissue&air*	*D_CTConoProbe*	*D_CTArtec*		*D_CTreproSurf*

^a^Acquisition protocol detailed in section Acquisition protocol

^b^Step detailed in section Evaluation of 'Surface to pseudo-CT study' conversion process

## Results

Table 2 summarises the details of the surface scanning with both devices. The laser power (ConoProbe) was manually increased in the configuration with blood owing to the absorption of the red laser light. The scanning process (of both scanning the surgical field and the markers) with ConoProbe took longer than with Artec, although the number of vertexes acquired with the latter was higher. Additional post-processing of Artec data (namely removal of noisy data, selection of surgical field and hole closing) was more laborious than that for ConoProbe data (namely outlier removal and selection of surgical field). The point-surface error (PSE, root-mean-square distance between the ConoProbe point set and its reconstructed surface) was around 2 mm (Table 2). This difference was not calculated for Artec since its output was a triangle mesh, not a point set.

**Table 2:** Acquisition data of both 3D scanning systems and registration results

	Tracked ConoProbe sensor						Artec scanner				*CTpostSurf*
	Laser power (%)	Scanning time (s)	No. of points	PSE^a^ (mm)	FRE^b^ (mm)	TRE^c^ (mm)	Scanning time (s)	No. of vertexes	FRE^b^ (mm)	TRE^c^ (mm)	RMSE^d^ (HU)
Without blood	20.3	731^e^	18,645	2.02	1.05	1.69 (11.23)	310	934,238	0.76	1.85 (12.82)	25
With blood	29.3	790^f^	25,076	1.96	1.11	1.51 (7.32)	339	757,887	0.70	1.44 (10.34)	20

^a^PSE (Point-surface error): root-mean-square distance between the ConoProbe point set and its reconstructed surface

^b^FRE (Fiducial registration error): root-mean-square distance between the markers in the 3D surface evaluated and in CTpreSurf

^c^TRE (Target registration error): root-mean-square distance between vertexes of the triangle mesh evaluated and vertexes of the 3D surface obtained from CTtissue&air. Maximum distance between those surfaces in brackets

^d^RMSE: root-mean-square error between CTpreSurf and CTpostSurf

^e^601 s and 130 s for scanning the surgical field and the markers respectively

^f^490 s and 300 s for scanning the surgical field and the markers respectively

In each configuration, *CTpostSurf* was superimposed to *CTpreSurf* to check the stability of the whole setting (surgical field and markers). Table 2 shows the RMSE between both studies without applying any alignment. The RMSE was lower than 26 HU in both configurations. In addition, the only difference found when comparing *CTpreSurf* in both configurations was the blood at the base of the surgical field. The position and orientation difference between the four CT studies was neglectable (lower than 0.30 mm and 0.05° respectively). These values were obtained from the transformation matrix calculated when mapping *CTpreSurf* (configuration with blood) and *CTpostSurf* (both configurations) to *CTpreSurf* (configuration without blood) with the landmark rigid registration algorithm explained in section Surface registration and the 3D coordinates of the markers.

**Fig. 3:**
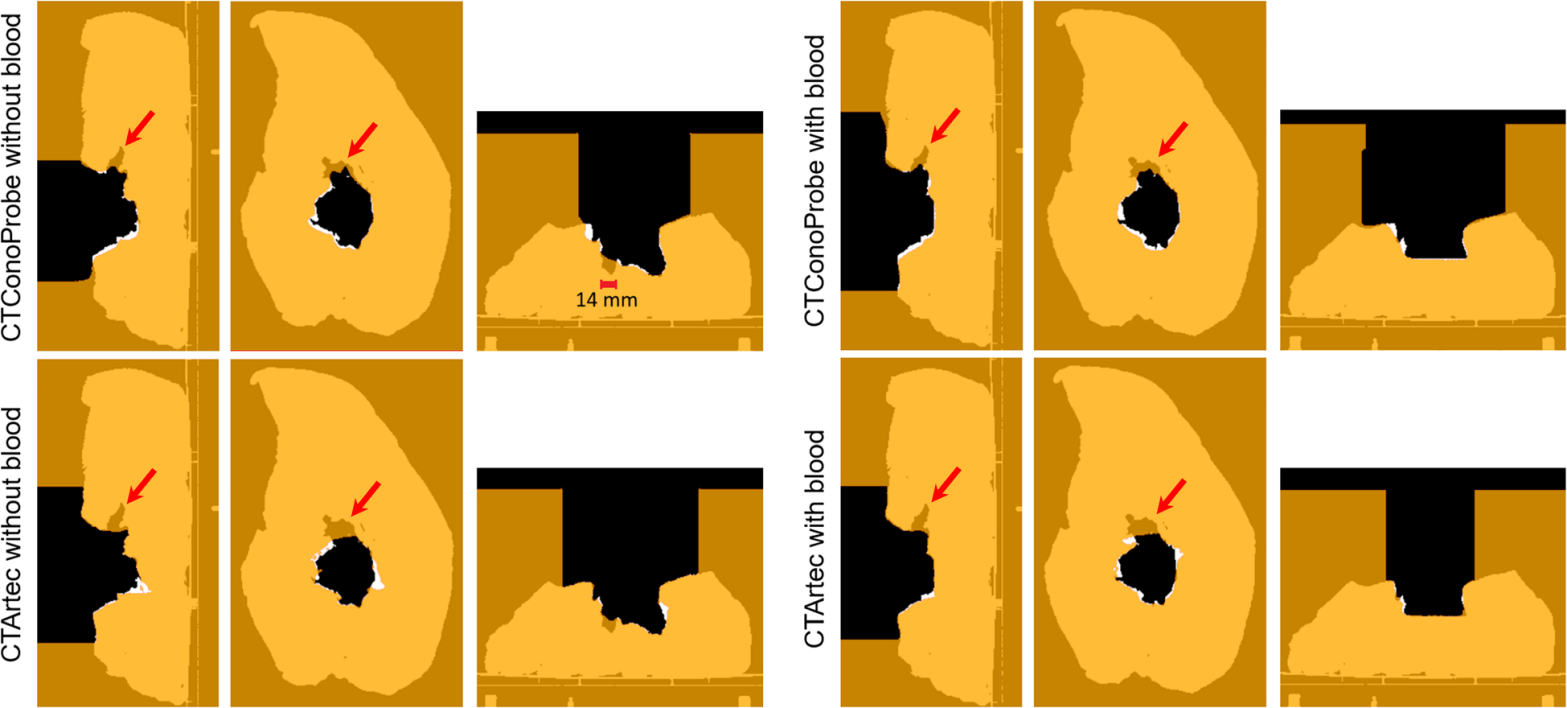
Comparison between *CTtissue&air* and the pseudo-CT studies *CTConoProbe* and *CTArtec* in both configurations (with and without blood). In this figure, each pseudo-CT study (tissue depicted in orange colour) was superimposed on *CTtissue&air* (tissue represented in white colour). In all cases, air was depicted in black colour. Therefore, tissue in the pseudo-CT study matches that in *CTtissue&air* when orange and white colours overlap. On the other hand, air in the pseudo-CT study matches that in *CTtissue&air* when black colour is shown in the figure. Other options represent mismatches. From left to right in each configuration, sagittal, coronal and axial views. The red arrows point to a deep hole not completely scanned

Table 2 shows the registration results between 3D surfaces and *CTpreSurf*. Target registration error (TRE, root-mean-square distance between vertexes of each triangle mesh and vertexes of the 3D surface obtained from *CTtissue*&*air*) was lower than 1.9 mm in all cases. Some holes inside the surgical field did not perfectly match when comparing *CTtissue&air*, and the pseudo-CT studies *CTConoProbe* and *CTArtec*, as depicted in Fig. 3.

**Fig. 4:**
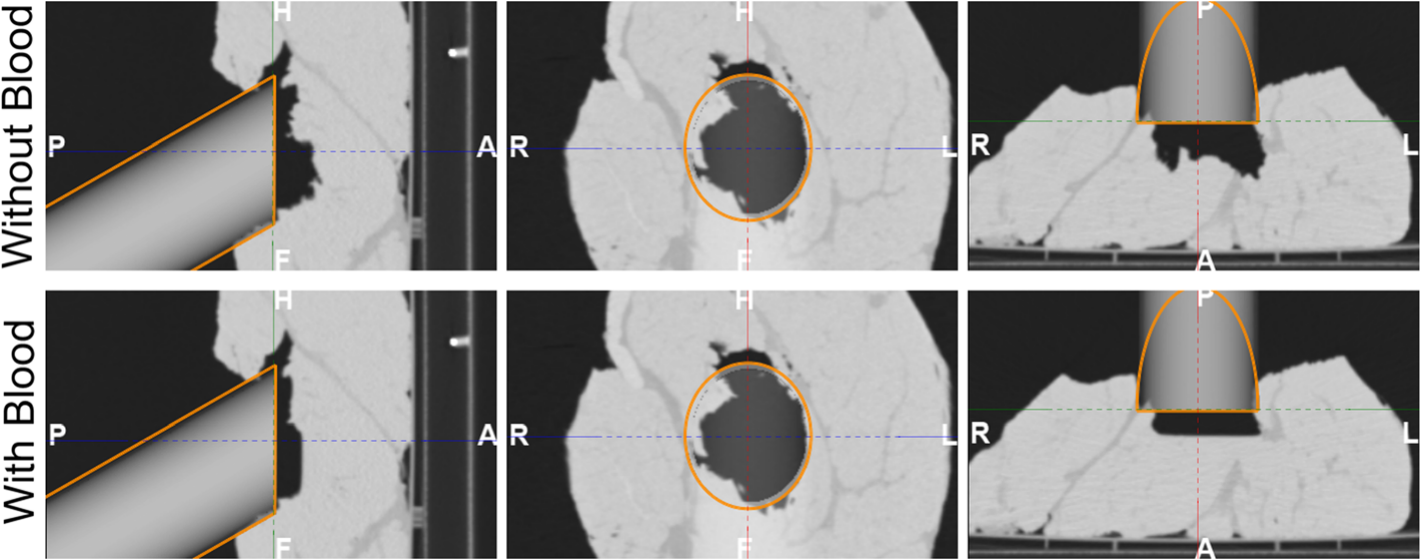
IOERT scenario in both configurations (with and without blood). Virtual applicator (grey cylinder with its contour in orange colour) superimposed on *CTpreSurf*. This figure shows the surface irregularities of the *irradiated* volume and the air gap from the applicator end to the simulated tumour bed. From left to right, sagittal, coronal and axial views. H (head), F (feet), A (anterior), P (posterior), R (right) and L (left)

**Fig. 5:**
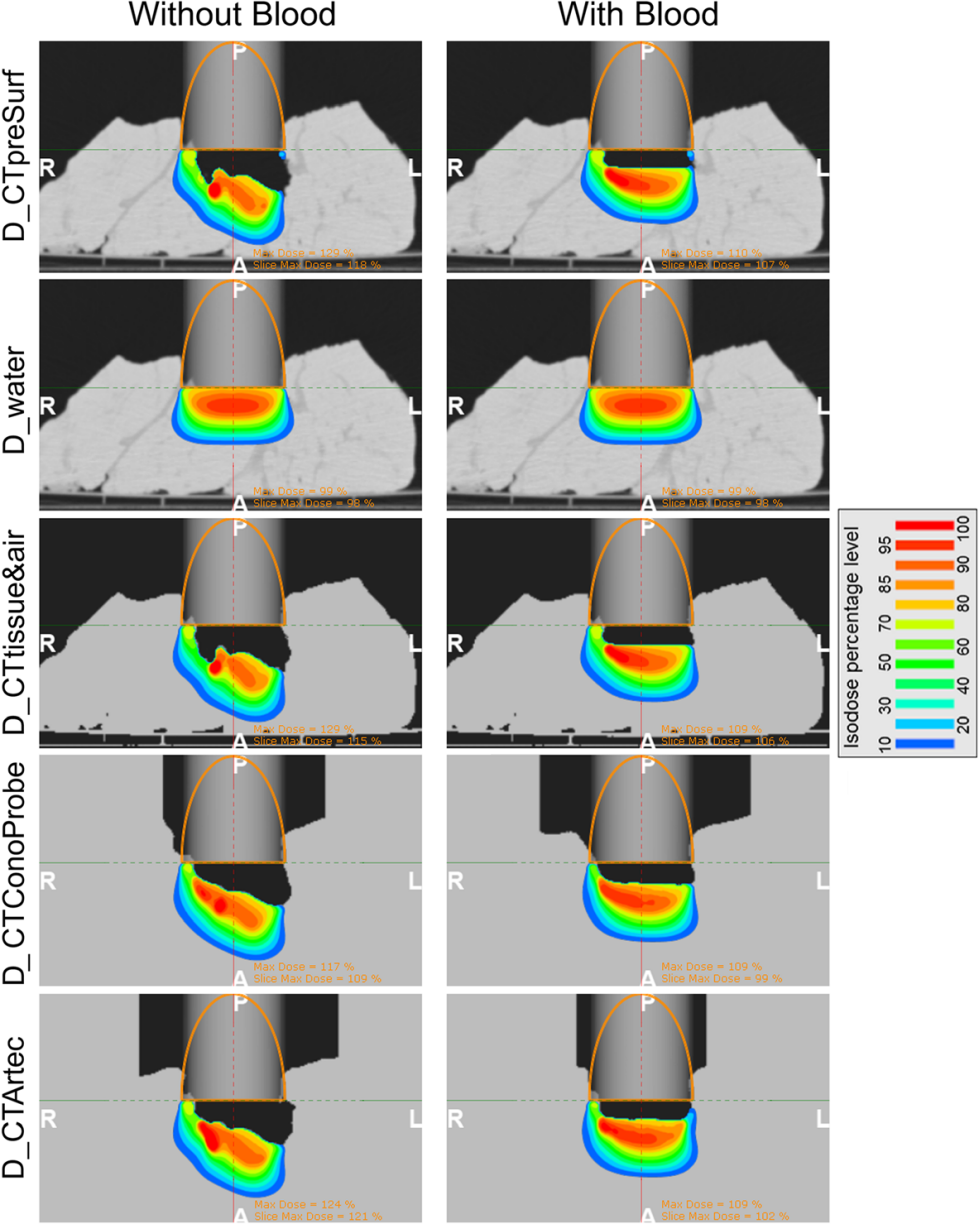
3D dose distributions calculated using the Monte Carlo algorithm (axial view at the bevel centre). Virtual applicator represented as a grey cylinder with its contour in orange colour. H (head), F (feet), A (anterior), P (posterior), R (right) and L (left)

**Fig. 6:**
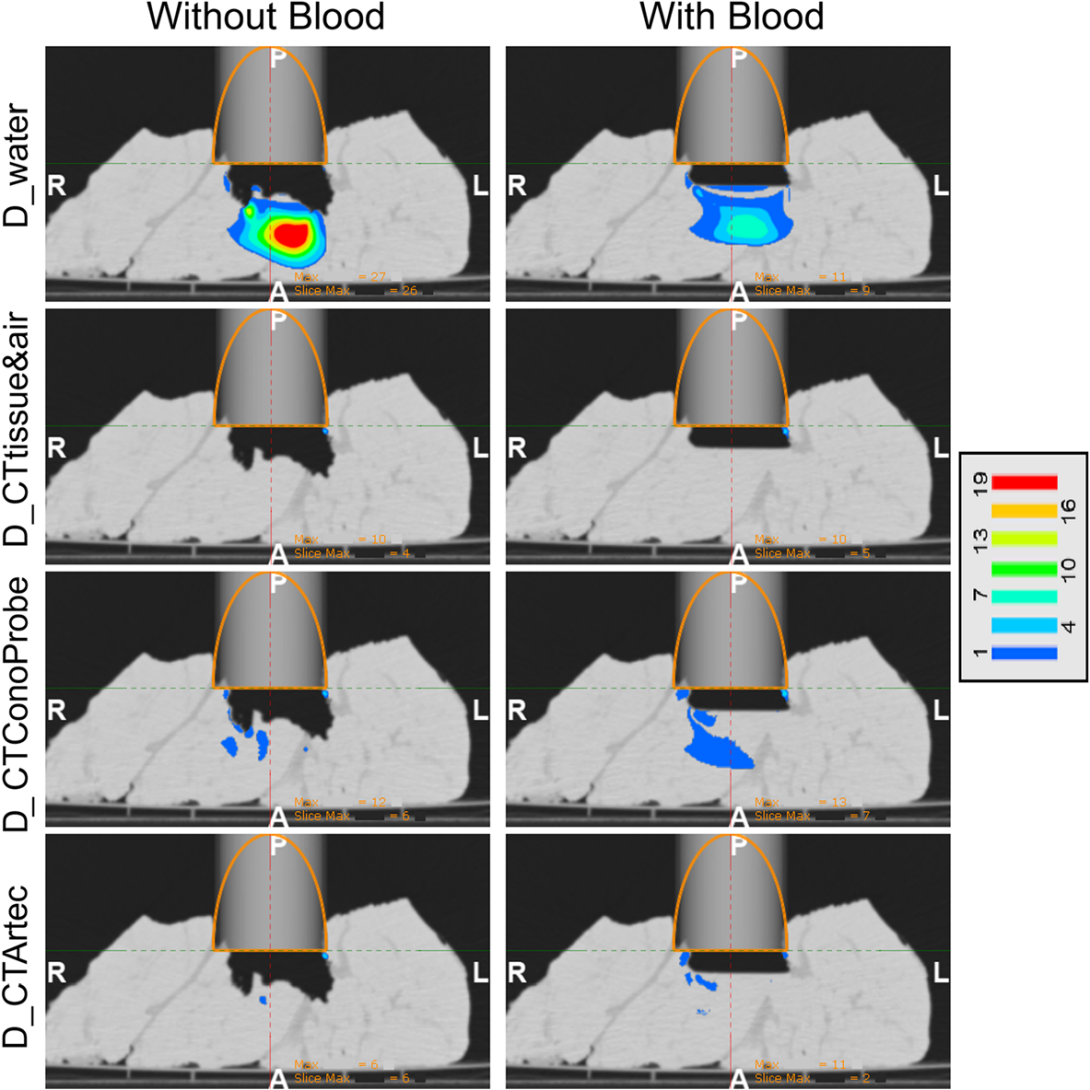
Gamma distributions superimposed on *CTpreSurf* (comparison for dose values greater than 10%). Gamma values lower or equal to 1 are not shown. Same axial view showed as that in Fig. 5

Figure 4 illustrates the complexity of the simulated tumour bed and the IOERT scenario set in each configuration. The maximum distance from the applicator end to the surface of the simulated tumour bed was 33.5 mm (configuration without blood) and 13.2 mm (configuration with blood). Dose distributions are shown in Fig. 5 and gamma pass rates are detailed in Table 3. *D_CTtissue*&*air* closely followed *D_CTpreSurf* in both configurations (average gamma pass rate 99.7% for dose values greater than 10%) while *D_water* differed from *D_CTpreSurf* (39.9%). A better dose agreement was found in the case of *D_CTConoProbe* (88.5%) and *D_CTArtec* (92.9%). Figure 6 shows the gamma distribution for the dose distributions evaluated to identify, in that axial view, where the gamma criteria failed in the comparison for dose values greater than 10%.

**Table 3:** Percentage of voxels fulfilling the 3D gamma criteria^a^

		3%/3 mm	
		Dose > 10%	Dose > 70%
Without blood	*D_CTpreSurf* v. *D_water*	36.5	28.0
	*D_CTpreSurf* v. *D_CTtissue&air*	**99.6**	**99.8**
	*D_CTpreSurf* v. *D_CTConoProbe*	**91.4**	84.3
	*D_CTpreSurf* v. *D_CTArtec*	**93.6**	88.2
With blood	*D_CTpreSurf* v. *D_water*	43.2	31.9
	*D_CTpreSurf* v. *D_CTtissue&air*	**99.8**	**99.9**
	*D_CTpreSurf* v. *D_CTConoProbe*	85.6	85.9
	*D_CTpreSurf* v. *D_CTArtec*	**92.1**	**90.9**

^a^Dose matrices: 151 × 298 × 320. Voxel size 1.0 × 1.0 × 1.0 mm. Gamma pass rates ≥90% are highlighted in bold

Average gamma pass rates for dose values greater than 10%: 39.9%, 99.7%, 88.5% and 92.9% for D_water, D_CTtissue&air, D_CTConoProbe and D_CTArtec respectively

**Fig. 7:**
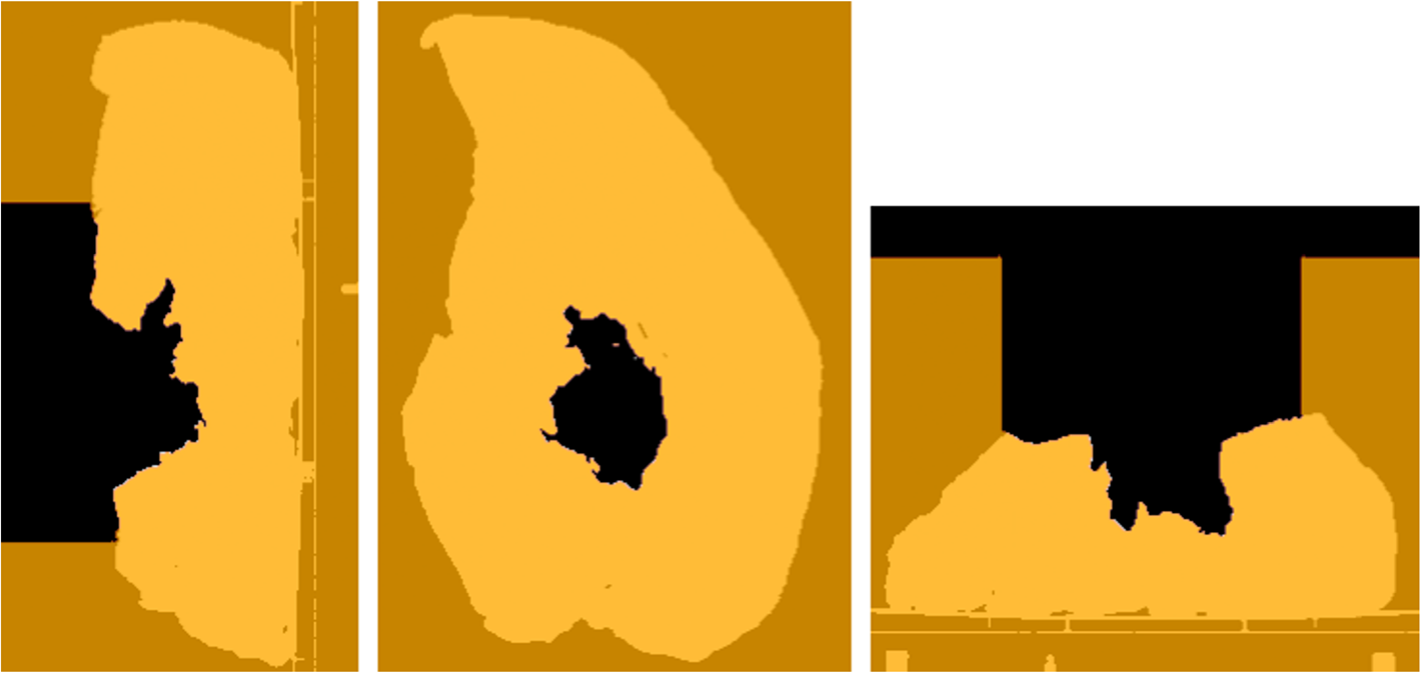
Comparison between *CTtissue&air* and the pseudo-CT study *CTreproSurf*. *CTreproSurf* (tissue depicted in orange colour) was superimposed on *CTtissue&air* (tissue represented in white colour). In both cases, air was depicted in black colour. More information on how to interpret the figure colours in Fig. 3

Figure 7 shows the agreement between the pseudo-CT studies *CTreproSurf* and *CTtissue*&*air*. These studies were used to assess the influence of the conversion from a 3D surface into a pseudo-CT study on the dose calculation. The percentage of voxels fulfilling the 3D gamma criteria when comparing *D_CTreproSurf* with *D_CTtissue*&*air* was 100.0% for dose values greater than 10% and for dose values greater than 70%.

## Discussion

Two different 3D scanning technologies were evaluated in the context of 3D dose calculation in IOERT. Both devices can scan at a distance greater than 30 cm (minimum safety margin between unsterile personnel and the sterile field [33]), suitable for the IOERT theatre. Advantages of Artec over ConoProbe are a larger working distance (up to 1 m) and a shorter scanning time. Nevertheless, ConoProbe can obtain better measurements inside narrow cavities since its laser beam can access them more easily than the structured 2D patterns generated by the Artec projector. However, neither device could completely scan the deeper hole of the simulated surgical field (Fig. 3, red arrows), although ConoProbe was slightly better in this task.

CT studies (namely *CTpreSurf* and *CTpostSurf*) were used to check the stability of the whole setting (surgical field and markers) and to obtain reference surfaces/studies and dose distributions. The results of the comparison between *CTpreSurf* and *CTpostSurf* showed a similar scenario in each configuration (with and without blood). In addition, a similar IOERT case was simulated in both configurations except for blood accumulation. The air gap of the configuration without blood (maximum distance from the applicator end to the surface of the simulated tumour bed 33.5 mm) was within the range seen in limb sarcomas (air gaps up to 5 cm [34]). However, the IOERT scenario simulated is not realistic for breast IOERT, where the smoothness of the irradiation surface and a minimum air gap from the applicator end to the tumour bed can be ensured by other means [35].

The use of surface scanning for 3D dose calculation in IOERT is based on assuming water-equivalent tissues at the surface of the tumour bed, not at the applicator end as in the conventional assumption. The gamma pass rates obtained with *D_CTtissue*&*air* validated this assumption for our experiment (average value 99.7% for dose values greater than 10%) and therefore lower gamma pass rates are due to surface defects in the pseudo-CT studies. This statement may not be valid when bone is included in the irradiation volume, as for instance in a rectal cancer case where the tumour bed or the high-risk area is very close to the sacrum.

In the simulated IOERT scenario, the conventional assumption of water-equivalent tissues at the applicator end led to inaccurate dose distributions in both configurations (average gamma pass rate 39.9% for dose values greater than 10%). The configuration with blood presented a different surface of the simulated tumour bed (irradiation surface flatter than that without blood) and a different air gap compared with the configuration without blood. Results improved when surfaces were considered in the dose calculation although there were regions in the surgical field that did not completely match, such as the 14-mm-width hole in the configuration without blood (Fig. 3, axial view of *CTConoProbe* and *CTArtec*). This hole could not be properly scanned since a small piece of tissue protruded and covered part of the hole. On the other hand, both devices could scan a surgical field with blood.

The match between *CTtissue*&*air* and the pseudo-CT studies *CTConoProbe* and *CTArtec* in the target volume was better in the case of Artec compared with ConoProbe (Fig. 3). Similarly, gamma pass rates with Artec were better than those with ConoProbe (average values 92.9% and 88.5% for dose values greater than 10% respectively). Possible sources of error in the ConoProbe measurements were the surface reconstruction from noisy points (PSE around 2 mm, Table 2) and larger error in the location of the markers compared with Artec (fiducial registration error [FRE] Table 2).

The pseudo-CT study *CTreproSurf* fitted the CT study used to generate *reproSurf* (namely *CTtissue*&*air*). These CT studies were utilised to assess the influence of the conversion from a 3D surface into a pseudo-CT study on the dose calculation. The results showed that the influence was negligible for the configuration without blood (the worst case of those presented in this study). Therefore, the error in the dose distributions *D_CTConoProbe* and *D_CTArtec* was caused by the inaccuracies of the scanned surfaces, and the registration between the 3D surfaces and *CTpreSurf*. 3D scanning from multiple viewpoints could improve the acquired data and thus increase the gamma pass rates, although it would further alter the surgical workflow.

Other studies have proposed in-vivo dosimetry with micro metal oxide semiconductor field-effect transistors, radiochromic films or thermoluminescence radiation detectors, which enables point and 2D dose measurements in IOERT [8, 36, 37]. Our approach focuses only on obtaining 3D dose distributions and some issues could still be improved. Applicator position and angle of beam incidence (applicator rotation) in relation to the patient’s anatomy are required for dose calculations. After scanning the surface of the tumour bed, the applicator position and rotation could be collected with an optical tracking system [38], and then combined with that scanned surface to also obtain the air gap from the applicator end to the surface of the irradiated volume. The applicator data would be in the same coordinate space as the scanned surface when using ConoProbe (same optical tracking system) while a calibration tool visible to the tracking system and the Artec scanner should be added to the scenario to map the applicator pose to the scanned surface. Finally, it would be necessary to reduce the data post-processing steps detailed in section Surface to pseudo-CT study for IOERT dose calculation and section Results in order to obtain a real-time implementation in the clinical practice, although this is not crucial since the main interest was to obtain the actual dose distribution for quality assurance of IOERT, but not immediately before irradiation.

## Conclusions

This is the first study that explores the use of surface scanning for 3D dose calculation in IOERT. In the simulated IOERT scenario, the conventional assumption of water-equivalent tissues at the applicator end led to inaccurate dose distributions. More accurate dose distributions were obtained when considering surfaces in the dose calculation of our simulated surgical field. The structured-light 3D scanner provided the best results in terms of dose distributions. Surface scanning is a promising method that could be easily included in the clinical practice, since the acquisition process is simple and does not require much time, and the calculated dose is comparable to the one that could be obtained with CT imaging. The findings obtained in this specific experimental setup warrant further research on surface scanning in the IOERT context owing to the clinical interest of improving the documentation of the actual IOERT scenario.

## Abbreviations

2D: Two-dimensional
3D: Three-dimensional
CT: Computed tomography
CTArtec: Pseudo-CT study obtained from Artec surface
CTConoProbe: Pseudo-CT study obtained from ConoProbe surface
CTpostSurf: CT study of the surgical field and the markers acquired after surface scanning
CTpreSurf: CT study of the surgical field and the markers acquired before surface scanning
CTreproSurf: Pseudo-CT study obtained from reproSurf
CTtissue&air: Pseudo-CT study obtained after converting CTpreSurf data to only two values, tissue and air
D_CTArtec: 3D dose distribution calculated from CTArtec
D_CTConoProbe: 3D dose distribution calculated from CTConoProbe
D_CTpreSurf: 3D dose distribution calculated from CTpreSurf
D_CTreproSurf: 3D dose distribution calculated from CTreproSurf
D_CTtissue&air: 3D dose distribution calculated from CTtissue&air
D_water: 3D dose distribution calculated under the conventional assumption of water-equivalent tissues at the applicator end
FRE: Fiducial registration error
HU: Hounsfield units
IOERT: Intraoperative electron radiation therapy
LINAC: Linear accelerator
PSE: Point-surface error
reproSurf: Surface generated from CTtissue&air
RMSE: Root-mean-square error
TPS: Treatment planning system
TRE: Target registration error
